# The role of pyroptosis in cognitive impairment

**DOI:** 10.3389/fnins.2023.1206948

**Published:** 2023-06-02

**Authors:** Xin Yang, Zhe Tang

**Affiliations:** ^1^Department of Oncology, Tongji Hospital of Tongji Medical College, Huazhong University of Science and Technology, Wuhan, China; ^2^Department of Thoracic Surgery, Tongji Hospital of Tongji Medical College, Huazhong University of Science and Technology, Wuhan, China

**Keywords:** pyroptosis, cognitive impaiment, NLRP3 inflammasome, treatment, GSDMD

## Abstract

Cognitive impairment is a major global disease, manifests as a decline in cognitive functioning and endangers the health of the population worldwide. The incidence of cognitive impairment has increased rapidly with an increasingly aging population. Although the mechanisms of cognitive impairment have partly been elucidated with the development of molecular biological technology, treatment methods are very limited. As a unique form of programmed cell death, pyroptosis is highly pro-inflammatory and is closely associated with the incidence and progression of cognitive impairment. In this review, we discuss the molecular mechanisms of pyroptosis briefly and the research progress on the relationship between pyroptosis and cognitive impairment and its potential therapeutic values, to provide a reference for research in the field of cognitive impairment.

## Introduction

Cognitive impairment is defined as a decline in cognitive functioning and endangers the health of the population especially elder people worldwide. The degree of cognitive impairment ranges from mild subjective cognitive impairment to severe dementia. Mild cognitive impairment (MCI) has a prevalence of approximately 6% in ages 60–64 and increases to approximately 25% in those ages 80–84 (Petersen et al., 2018). Approximately 5%–10% of people with MCI progress to dementia annually (Sanford, 2017). With an increasingly aging population, the incidence of cognitive impairment has increased rapidly. Although the mechanisms of cognitive impairment have partly been elucidated with the development of molecular biological technology, however, the tools and strategies for the treatment of cognitive impairment remained limited. To fully understand the neuropathological changes of cognitive impairment and develop effective therapeutic methods, there is still a long way to go.

The long-recognized modes of cell death are limited to apoptosis and necrosis. Apoptosis is mainly characterized by cell shriveling and the formation of apoptotic bodies, which are then rapidly engulfed by surrounding phagocytes without causing an inflammatory response. Necrosis was previously thought to be an unregulated and passive death process, but with further research, it has now been shown that some of the necrosis can be controlled and is called programmed necrosis (Tonnus et al., 2019), of which pyroptosis is one of the main forms. Recently increasing studies have revealed that pyroptosis played a significant role in cognitive impairment. This review summarized the molecular mechanisms of pyroptosis and focus on the function of pyroptosis involved in the pathogenesis and treatment of cognitive impairment-related diseases.

## Molecular mechanisms of pyroptosis

**Figure 1:**
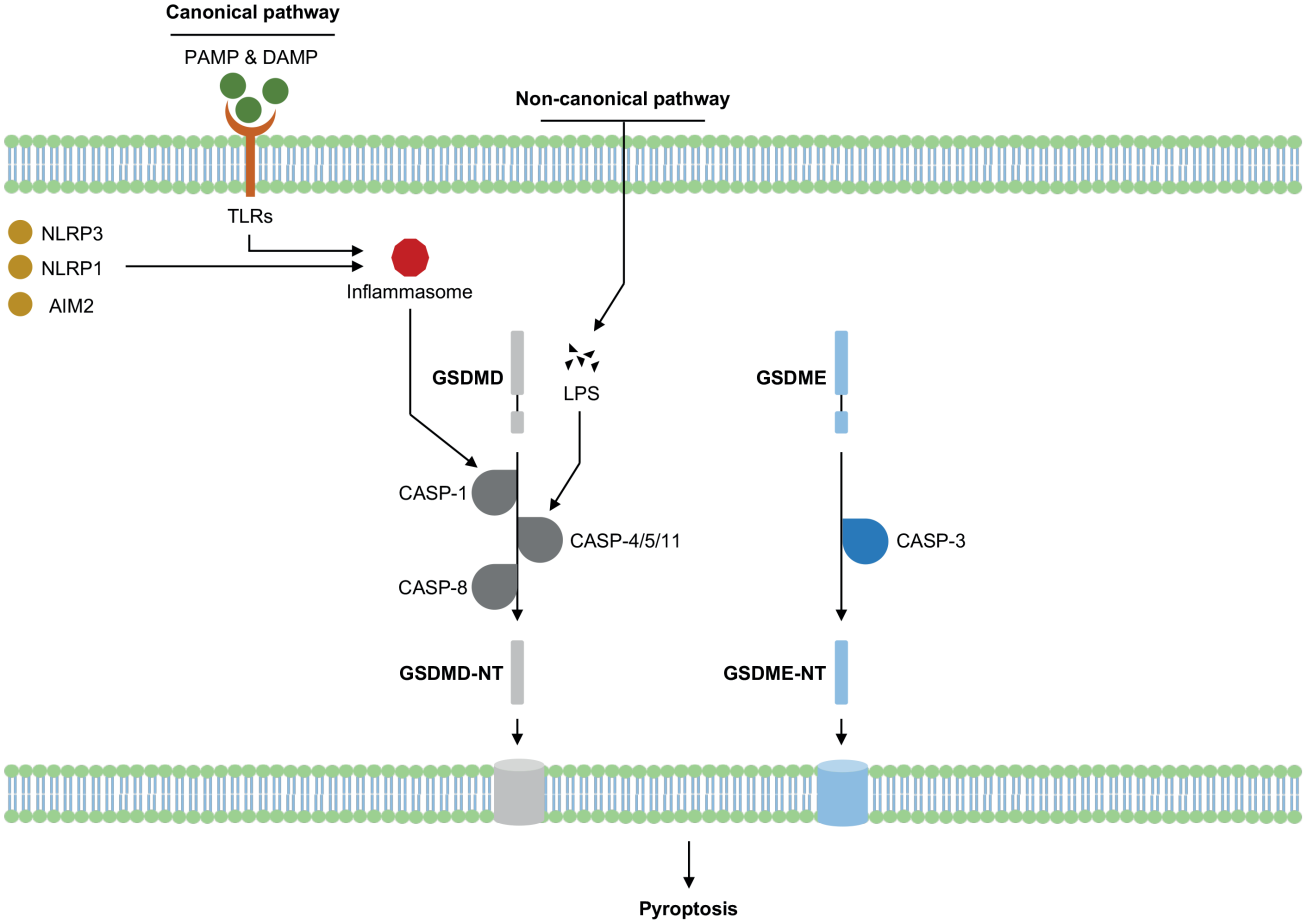
Summary of pyroptosis activation in cognitive impairment-related diseases. LPS, Lipopolysaccharide; CASP, Caspase; GZM, Granzyme; NT, N-terminal domain; PAMP, Pathogen-associated molecular patterns; DAMP, Damage-associated molecular patterns; TLRs, Toll-like receptors; NLRP3, nucleotide-binding oligomerization domain-like receptor protein 3; NLRP1, NLR family pyrin domain containing 1; AIM2, melanoma deficiency factor 2.

Pyroptosis, a highly pro-inflammatory programmed cell death, was first observed in macrophages after a bacterial infection or treatment with bacterial toxins and was for a long time mistaken for a macrophage-specific cell death dependent on caspase-1, a pro-inflammatory protease cleaving interleukin 1β (IL-1β) (Thornberry et al., 1992). Subsequent studies revealed that intracytoplasmic pattern recognition receptors (PRRs) recognized exogenous pathogen-associated molecular patterns (PAMPs) or endogenous dangerous signaling to form inflammasomes that recruited and activated caspase-1, thus leading to pyroptosis (canonical pyroptosis pathway); murine caspase-11 and human caspase-4/5 could act directly as PRRs to recognize inflammasome assembled by polysaccharide-like lipid A and also result in pyroptosis (non-canonical pyroptosis pathway), which overturned the traditional concept of inflammasome (Fang et al., 2020). Recent studies have found that caspase-1 and caspase-11/4/5 both cleaved the common substrate gasdermin D (GSDMD) and led to pyroptosis (Kayagaki et al., 2015; Shi et al., 2015), gasdermin family proteins were identified as key effector molecules that mediate the onset of pyroptosis (Figure 1).

The canonical pyroptosis pathway was dependent on the activation of caspase-1, which exerted its effects via the inflammasome pathway. Inflammasome was first proposed in 2002 (Martinon et al., 2002) and was now defined as a class of multimeric protein complexes mainly composed of PRRs, apoptosis-associated speck-like protein containing CARD (ASC), and pro-caspase-1, to identify various irritating and damaging signals in natural immune responses and had a close relationship to the occurrence of cell death (Lamkanfi and Dixit, 2017). PRRs mainly included Toll-like receptors (TLRs), melanoma deficiency factor 2 (AIM2) and NOD-like receptors (NLRs), of which nucleotide-binding oligomerization domain-like receptor protein3 (NLRP3) got the most attention. PRRs could identify PAMPs or endogenous dangerous signaling to form inflammasome complex after two stages of priming and activation, which in turn activates its downstream caspase-1. Activated caspase-1 could cleave GSDMD to GSDMD-N terminal, forming pores in the cell membrane. Meanwhile, the pro-inflammatory factors pro-IL-1 and pro-IL-18 were also cleaved by activated caspase-1, transferred to mature IL-1β and IL-18, releasing from the cell membrane pores, thus inducing pyroptosis. Numerous studies have demonstrated that NLRP3/caspase-1 mediated pyroptosis signaling pathway played a crucial role and was the most common therapeutic target of cognitive impairment (Van Zeller et al., 2021; Li et al., 2022), any blockade of NLRP3/caspase-1 mediated pyroptosis may reverse the incidence or progression of cognitive impairment.

The non-canonical pyroptosis pathway was dependent on the activation of human caspase-4/-5 and homologous murine caspase-11 by intracellular lipopolysaccharides (LPS). LPS typically consisted of a hydrophobic domain known as lipid A (or endotoxin), a non-repeating “core” oligosaccharide, and a distal polysaccharide (or O-antigen) (Raetz and Whitfield, 2002). LPS could directly bind and activate caspase-4/-5/-11 protein to initiate pyroptosis (Zheng et al., 2021). Similar to the canonical pyroptosis pathway, activated caspase-4/-5/-11 cleaved GSDMD to GSDMD-N terminal, inducing pore formation in the cell membrane (Shi et al., 2017). During the process, LPS could also induce caspase-11-dependent cleavage of the pannexin-1 channel protein, activating the P2X purinoreceptor 7 (P2X7) receptor-dependent membrane pore opening and causing subsequent ATP release, K^+^ efflux, osmotic imbalance, leading to cell swelling and membrane rupture, and eventually resulting in pyroptosis (Shi et al., 2015; Liu et al., 2016; He et al., 2023). Furthermore, released ATP and K^+^ efflux through pannexin-1 transmembrane channel could activate NLRP3 inflammasome and IL-1β secretion, indicating that NLRP3 might be a crucial bridge between the canonical and non-canonical pyroptosis pathways (Karmakar et al., 2015; Yang et al., 2015; Karmakar et al., 2016).

GSDMD belonged to gasdermin family proteins and was primarily identified by two separate studies in 2015, Dixit et al. found that GSDMD played a key role in LPS-induced activation of the non-canonical inflammasome by screening chemically induced mouse mutants (Kayagaki et al., 2015), while Shao et al. performed a genome-wide screen of caspase-11 and caspase-1-induced pyroptosis pathways in cell lines, which revealed that GSDMD is the substrate of all inflammatory caspases and is the true executor of pyroptosis (Shi et al., 2015). Multiple caspases such as caspase-1/-4/-5/-8/-11 could cleave and activate GSDMD. Caspase-1 activated GSDMD through inflammasome complexes such as AIM2, NLRC4 or NLRP3 (Broz and Dixit, 2016). The activation of GSDMD by caspase-4 was reported to be regulated by interferon regulatory factor 2 (Benaoudia et al., 2019; Kayagaki et al., 2019). It was reported that caspase-8-dependent GSDMD cleavage relied on caspase-8 dimerization and autoprocessing (Demarco et al., 2020). LPS-triggered caspase-11-GSDMD signaling pathway was upregulated by IFN-γ and IFN-β (Brubaker et al., 2020; Zhang et al., 2020). Unlike GSDMD-mediated pyroptosis, gasdermin E (GSDME)-induced pyroptosis mainly relied on the activation of caspase-3. Traditionally caspase-3 is an apoptosis-related caspase, which could be activated under the treatment of TNF-α or chemotherapy drugs, inducing cell apoptosis. However, when GSDME existed, activated caspase-3 would cleave GSDME at the site of residue Asp270 and induce pyroptosis instead of apoptosis (Ouyang et al., 2023). The role of GSDMD and GSDME-mediated pyroptosis in cognitive impairment have been elucidated, but the mechanisms of other gasdermin family members such as GSDMA, GSDMB, GSDMC and DFNB59 in cognitive impairment remained unclear.

## Pyroptosis and cognitive impairment

### Alzheimer’s disease

The most common neurodegenerative condition affecting the aged population is Alzheimer’s disease (AD), which is characterized by a particular sequence of pathological alterations in the brain that cause neurodegeneration, loss of synaptic connections, progressive memory problems, and cognitive impairment (Masters et al., 2015). Unfortunately, despite a large number of studies on the mechanisms of AD, there were no approved therapies to halt or reverse its progression (Van Zeller et al., 2021). Recent studies elucidated the relationship between pyroptosis and AD, and some drugs targeting the pyroptosis pathway showed therapeutic potential.

Rui et al. (2021) detected the expression levels of NLRP3, caspase-1, GSDMD, and IL-1β and found all the above parameters were increased in the peripheral blood mononuclear cells (PBMCs) of amnestic mild cognitive impairment (aMCI) and AD patients, and IL-1β was positively associated with the disease, indicating the important role of pyroptosis in AD. Li et al. (2020) found that neuronal pyroptosis induced by the overexpression of NLRP3/caspase-1/GSDMD axis was the key cause of neuronal loss in AD, NLRP3 inhibitor MCC950 could inhibit neuronal pyroptosis by downregulating NLRP3/caspase-1/GSDMD axis, reduced the neurotoxicity of amyloid-β_1-42_ (Aβ_1-42_) *in vitro*, improved the spatial memory ability *in vivo*. NLRP3 inhibitor might be a potential therapeutic agent of AD. Tian et al. (2021) revealed that activated caspase-1 directly induced pyroptosis through NLRP3 and AIM2 activation in an AD mouse model induced by sevoflurane, caspase-1 small-molecule inhibitor VX-765 could significantly inhibit the pyroptosis pathway, suppress the release of IL-1β and IL-18 and downregulate tau phosphorylation, thus restoring neuron function of AD. Several studies focused on the ameliorative effects of traditional Chinese medicine in AD. Salidroside, the main pharmacological active ingredient isolated from *Rhodiola rosea L*. (Cai et al., 2021), Dendrobium nobile Lindl. Alkaloid (DNLA), the main active compound in *Dendrobium nobile Lindl* (Li et al., 2022), as well as Jiedu-Yizhi formula (Wang et al., 2022) were found to inhibit NLRP3-mediated or LPS-induced pyroptosis and improve AD. Li et al. (2021) revealed that Schisandrin, a representative lignan of *Schisandra chinensis Bail*., could also inhibit Aβ-induced NLRP-1-mediated neuronal pyroptosis and ameliorate cognitive impairment of AD. Quinones in Chinese Medicine could potentially prevent AD via modulating the NLRP3 inflammasomes, adopt molecular docking study indicated that purpurin and rhein might be the most promising NLRP3 inhibitors, however, further study was required to ascertain the preventive effect (Chen et al., 2020). Bai et al. (2021) elucidated that N-salicyloyl tryptamine derivatives could restore Aβ-induced pyroptosis through NLRP3/caspase-1/GSDMD axis and ameliorate cognitive function. It was reported that upregulated RAGE–TXNIP axis or activated transient receptor potential vanilloid 4 (TRPV4) participated in causing pyroptosis and result in cognitive impairment in AD, which could be the potential therapeutic target through suppression of overactivated pyroptosis (Sbai et al., 2022; Guo et al., 2023).

### Perioperative neurocognitive disorder

Perioperative neurocognitive disorder (PND) is one of the common complications during the perioperative period and is mainly manifested as cognitive impairment. PNDs include acute postoperative delirium and relatively long-lasting postoperative cognitive dysfunction (Li et al., 2022). Age, surgical trauma, and anesthetics are the main risk factors of PND, however, the mechanisms of PND remained unclear. Many studies have elucidated the important role of pyroptosis in the pathogenesis of PND and targeting pyroptosis could be an effective method for PND treatment.

It was reported that NLRP3 inflammasome-mediated pyroptosis directly led to cognitive impairment in PND mice model induced by isoflurane, and NLRP3 inflammasome inhibitor MCC950 could inhibit overactivated pyroptosis and exert a neuroprotective effect, thus improving cognitive impairment (Fan et al., 2018). Zhou et al. demonstrated that pyroptosis, as well as the level of reactive oxygen species (ROS) was significantly upregulated in a postoperative cognitive dysfunction (POCD) mouse model induced by sevoflurane. Not only the pyroptosis inhibiter, necrosulfonamide (NSA), could improve cognitive impairment via suppressing pyroptosis, but also the ROS scavenger, N-acetylcysteine (NAC), could ameliorate POCD by reducing the level of ROS and pyroptosis through NLRP3 inflammasome pathway (Zhou et al., 2023). Caspase-1 inhibitor, VRT-043198, was reported to ameliorate PND in an aged mice model by inhibiting caspase-1-mediated pyroptosis (Tang et al., 2022). Zuo et al. (2020) revealed that elamipretide could also attenuate pyroptosis by inhibiting NLRP3/caspase-1 pathway and partly restore PND in aged mice. Que et al. (2020) demonstrated that isoflurane exposure resulted in cognitive dysfunction in aged rats, accompanied by decreased expression of DUSP14, and it was found that DUSP14 could regulate NLRP3-mediated pyroptosis, overexpression of DUSP14 inhibited pyroptosis and improved cognitive impairment, indicating that DUSP14 might be a new therapeutic target for POCD. Isoflurane induced hippocampal neuronal damage and cognitive impairment by upregulating SETD7 and activating pyroptosis in the hippocampus, knockdown of STED7 could inhibit the level of pyroptosis and the release of inflammatory cytokines, prevent hippocampus damage and improve POCD (Ma et al., 2022). Apart from NLRP3/caspase-1 axis, other pyroptosis pathways were revealed to be associated with PND. Wang et al. found that caspase-3 was activated in POCD, and activated caspase-3 could cleave gasdermin E (GSDME) to form GSDME-N terminal, forming pores in the cell membrane and inducing pyroptosis. Caspase-3 inhibitor Ac-DEVD-CHO (Ac-DC) could inhibit pyroptosis and improve cognitive impairment (Wang et al., 2023). NF-κB and HMGB1 induced pyroptosis were also elucidated in PND, NF-κB inhibitor or HMGB1 inhibitor treatment effectively improved PND by significantly inhibiting pyroptosis (Dai et al., 2021; Shan et al., 2023). Li et al. (2022) found that esketamine improved POCD in aged rats and alleviated the pyroptosis of astrocytes after LPS exposure, moreover, an underlying connection between STING/TBK1 signaling pathway and caspase-1-mediated pyroptosis was indicated, which required further research. MicroRNA-140-3p was found to improve POCD by repressing neuron pyroptosis via HTR2A/ERK/Nrf2 axis by targeting DNMT1 (Wu et al., 2022).

### Sepsis-associated encephalopathy

Sepsis-associated encephalopathy (SAE) is a frequent complication that leads to long-term cognitive impairments and psychiatric diseases in sepsis patients and has a close association with increased morbidity and mortality. The potential mechanisms of SAE are complex, including endothelial dysfunction, damage to the blood–brain barrier, oxidative stress, etc. However, the molecular changes in SAE required further research. Accumulating evidence has indicated that pyroptosis may be the bridge between SAE and overactivated neuroinflammation, especially the NLRP3/caspase-1 pathway. NLRP3 inhibitor MCC950 and the caspase-1 inhibitor Ac-YVAD-CMK or VX765 were used for the treatment of SAE and the results were exhilarating. Administration of the above inhibitors could repress overactivated pyroptosis and the release of pro-inflammatory cytokines, restore the synapse plasticity and preserve long-term potential, thus improving cognitive dysfunction (Fu et al., 2019; Xu et al., 2019). It was mentioned that electroacupuncture could improve 7-day survival rates and cognitive function by downregulating NLRP3/caspase-1/GSDMD pyroptosis pathway in an SAE mouse model (Li et al., 2022). As the most important pyroptosis pathway, the activity of NLRP3/caspase-1 signaling pathway was mediated by various cell components or molecules. Zhou et al. found that p38 MAPK and ERK signaling pathways might regulate NLRP3/caspase-1 pathway, and the phosphorylation of p38 MAPK and ERK was positively correlated with NLRP3, caspase-1, and inflammatory factor levels in SAE. Downregulation of p38 MAPK and ERK led to suppression of pyroptosis, however, the direct connection between these two pathways needed deeper exploration (Zhou et al., 2019). Jing et al. revealed the importance of IRE1α/Xbp1s-Ca^2+^ signaling in endoplasmic reticulum (ER) stress, which was involved in NLRP3 inflammasome activation. IRE1α/Xbp1s pathway was activated, promoting the ER Ca^2+^ influx to the cytoplasm and inducing NLRP3 inflammasome-mediated pyroptosis. The selective inhibitor STF083010 targeting IRE1α/Xbp1s could partly restore the process, and improved cognitive function by attenuating microglial pyroptosis (Jing et al., 2022). Mitochondria-mediated pyroptosis was reported in SAE, mitochondria impairment was associated with cognitive dysfunction. The mitochondrial protectant dexpramipexole (DPX) could sustain mitochondrial function and inhibit NLRP3/caspase-1 pyroptosis pathway, thus ameliorating neuroinflammation and cognitive impairment in SAE (Zhang et al., 2023). Sevoflurane was reported to activate HMGB1-induced NLRP3/ASC inflammasome, induce pyroptosis, and impair cognitive function in PND (Shan et al., 2023), however, in SAE, sevoflurane could act as protective role. Chen et al. (2022) demonstrated that sevoflurane could improve cognitive dysfunction by inhibiting the NLRP3-dependent caspase-1/11-GSDMD pathway, in which SIRT1 played a key role. Yang et al. showed that cannabinoid type 2 receptor (CB2R) helped to protect neurons and promote survival in SAE patients. Furthermore, it has been proven that the CB2R-specific agonist HU308 could repress neuronal pyroptosis, attenuate brain tissue damage and improve cognitive impairment in SAE (Yang et al., 2022).

### Cerebrovascular diseases

Cerebrovascular diseases are also key contributors to the overall burden of cognitive impairment and mainly include acute ischemia stroke and chronic cerebral hypoperfusion (CCH). An acute ischemic stroke occurs due to the sudden interruption or reduction of blood supply in part of the brain, and the process is often combined with pre-existing microvascular and neurodegenerative changes, which results in a series of pathological changes leading to cognitive impairment (Rost et al., 2021). CCH is caused by chronic reduction of cerebral blood flow, which is a common pathophysiological process in cerebral vascular diseases such as atherosclerosis or arteriosclerosis, leading to a state of prolonged ischemia and hypoxia in the brain tissue, finally results in progressive and persistent cognitive impairment (He et al., 2023). Although the mechanisms of cerebrovascular disease-induced cognitive dysfunction have not been fully understood, it has been identified that pyroptosis was involved in the pathological process.

Kim et al. elucidated that AIM2 inflammasome, as well as caspase-1, IL-1β, IL-18, was significantly upregulated in the hippocampus and cortex in the mouse model of post-stroke cognitive impairment than in those of the sham group. AIM2 inflammasome-mediated pyroptosis could cause acute and chronic neuronal death after stroke, which might result in cognitive dysfunction. Moreover, knockout of AIM2 or inhibition of caspase-1 could improve cognitive function and partly reverse brain volume in the hippocampus compared to those in stroke mice (Kim et al., 2020). Furthermore, Kim et al. developed a miniaturized electronic device of photobiomodulation (PBM), consisting of packaged light-emitting diodes (LEDs) that incorporate a flexible substrate for *in vivo* brain PBM in a mouse model. The preventive and therapeutic effects of PBM affixed to the exposed skull of stroke mice model were evaluated, and the results showed that the PBM with 630 nm LED array could significantly attenuate the progression of cognitive impairment in the chronic poststroke phase via regulating AIM2 inflammasome activation and AIM2 inflammasome-mediated pyroptosis (Kim et al., 2022). It was reported that NLRP3/caspase-1-dependent pyroptosis participated in the cerebral ischemia/reperfusion injury and cognitive decline after focal cortical infarction, NLRP3 inhibitor and caspase-1 inhibitor could improve the symptoms, respectively (Sun et al., 2020; Dong et al., 2022). The antiepileptic drug valproic acid (VPA) was also reported to improve cerebral ischemia/reperfusion injury via modulating an apoptosis repressor with caspase recruitment domain (ARC)-mediated caspase-1-dependent pyroptosis pathway (Zhu et al., 2019).

The activation of AIM2 inflammasome contributed to the pathophysiology of chronic CCH-induced brain injury (Poh et al., 2021), and it was revealed and knockout of AIM2 attenuated pyroptosis in the cerebellum following CCH mainly by decreasing the production of proinflammatory cytokines (Poh et al., 2021). It was reported that curcumin and emodin could protect against CCH-induced cognitive dysfunction via inhibiting overactivated NLRP3-dependent pyroptosis (Zheng et al., 2021; Jiang et al., 2023). Chai et al. found that the level of legumain, a lysosomal cysteine protease, was significantly increased in the hippocampus of mice with CCH, considering the abnormal upregulation of legumain in mediating synaptic plasticity impairment and neuroinflammation, targeting legumain might be a potential therapy for CCH. Legumin knockout could partly restore synaptic plasticity and protect against cognitive impairment by decreasing the levels of inflammatory cytokines and the inflammasome complex and inhibiting pyroptosis (Chai et al., 2021). Interestingly, behavioral therapy also showed potential for the treatment of CCH-induced cognitive dysfunction. Poh et al. (2021) observed increased expression of inflammasome components and precursor IL-1β in the brain tissue following CCH, intermittent fasting (16 h food deprivation daily) could significantly reduce the expression levels of cleaved caspases-1/-8/-11 and maturation of both IL-1β and IL-18, inhibit proptosis and improve cognitive impairment, suggesting the therapeutic effect of non-pharmaceutical intervention.

### Metabolic disorders

It has been well-recognized that metabolic disorders such as type 2 diabetes mellitus, obesity and cardiovascular diseases are associated with cognitive impairment (Zilliox et al., 2016; Sharma, 2021). Considerable molecular biological studies have elucidated the mechanisms of how diabetes and obesity/high-fat diet (HFD) caused cognitive impairment, among which pyroptosis played an important role.

Diabetes mellitus induced the risk and promoted the development of cognitive dysfunction mainly via the greater occurrence of small-/micro-vascular diseases or even stroke. Ward et al. found that diabetes could induce neuronal degeneration and blood–brain barrier disruption, thus impairing cognitive function. In the process amplified NLRP3 activation was observed, and NLRP3 inhibitor could ameliorate cognitive function and vascular integrity in a high-fat diet/streptozotocin-induced (HFD/STZ) diabetic male Wistar rat model with stroke. Although the role of pyroptosis was not deeply discussed, NLRP3 inhibitor showed therapeutic potential (Ward et al., 2019). Ruan et al. showed the effect of HECT domain E3 ubiquitin protein ligase 3 (HECTD3) in diabetes-related cognitive impairment. HECTD3 was upregulated, together with the increased levels of NLRP3/caspase-1/GSDMD pyroptosis pathway, in the hippocampus of STZ-induced diabetic rats and PC12 cells treated with high glucose medium. HECTD3 silencing could inhibit the activation of NLRP3 inflammasome, suppressed pyroptosis level and exerted a neuroprotective effect via MALT-mediated JNK signaling (Ruan et al., 2022). It was revealed that the P2X7-mediated NLRP1/Caspase-1 pyroptosis pathway, as well as apoptosis and oxidative stress, was overactivated in high glucose-induced hippocampal neuron injury. Naofucong, a compound preparation based on traditional Chinese medicine theory and modern pharmacology, was found to reduce both oxidative stress and pyroptosis by suppressing P2X7/NLRP1/caspase-1 pathway, finally improving cognitive impairment (Jing et al., 2021). Wang et al. elucidated the mechanism of how tetracyclic oxindole alkaloid isorhynchophylline (IRN) helped lessen diabetes-induced cognitive impairment. Spliced form of X-box binding protein 1 (sXBP1) played a crucial role in the process. IRN promoted sXBP1 translocation into the nucleus, and restored downstream high glucose-mediated impairment of insulin signaling, endoplasmic reticulum stress, and pyroptosis/apoptosis, thus improving cognitive dysfunction (Wang et al., 2023). Gestational diabetes mellitus (GDM) is defined as diabetes diagnosed for the first time during pregnancy and can lead to cognitive impairment in offspring. Liang et al. found that chemerin was significantly upregulated in the serum, placenta tissue, and umbilical cord blood of the diabetic mother, further study revealed that chemerin-induced diabetic pregnant disease via chemokine receptor-like 2 (CCRL2)-dependent enrichment of chemerin in the brain of offspring, which led to macrophage recruitment, activation of NLRP3/caspase-1 mediated pyroptosis, resulting in cognitive impairment. Chemerin exerted effects via chemerin receptor 23 (ChemR23), therefore targeting CCRL2 and ChemR23 could be effective for treating cognitive dysfunction in offspring of GDM (Liang et al., 2019).

Compared with cardiovascular diseases and diabetes mellitus, cognitive impairment is easy to be ignored in patients with obesity (Wang et al., 2017; Zhang et al., 2020). It is important to uncover the relationship between obesity/HFD and cognitive impairment. Sui et al. reported that under HFD conditions, neuronal pyroptosis was significantly increased, tau protein was hyperphosphorylated, Nrf-2/HO-1 signaling pathway was activated. Exogenous IGF-1 could improve cognitive impairment in a C57BL/6 J mice model fed with HFD by reversing the activity of the above signaling (Sui et al., 2021). MicroRNAs also showed potential in treating obesity/HFD-related cognitive impairment. Wang et al. found that HFD caused cognitive impairment following neuronal pyroptosis and a decrease of IGF-1/GSK3β signaling pathway in the midbrain and hippocampus tissues. Inhibition of miR-129 by miR-129 antagomir could attenuate NLRP3/caspase-1 mediated pyroptosis and improve cognitive impairment by activating IGF-1/GSK3β signaling pathway via directly targeting IGF-1 (Wang et al., 2021). Yang et al. used BV2 cells treated with palmitic acid to establish an *in vitro* model of HFD, the results showed TLR4/MyD88/NF-κB p65 signaling, together with NLRP3 expression, was upregulated in palmitic acid-treated BV2 cells. miR-124 could target TLR4/MyD88/NF-κB p65/NLRP3 signaling and reduce its activity, showing a protective effect against HFD-induced neuronal injury (Yang et al., 2022).

## Discussion and perspectives

Although the effects and mechanisms of pyroptosis in cognitive impairment are not fully understood, numerous pieces of evidence showed that the various pyroptosis signaling pathway participated in the incidence and progression of cognitive dysfunction induced by different causes. Pyroptosis was regulated by canonical, non-canonical and gasdermin-dependent signaling pathways, forming a complex regulation network. The levels of pyroptosis signaling pathway were significantly upregulated in cognitive impairment, together with the release of large amounts of inflammatory substances such as IL-1β and IL-18, resulting in a cascade of inflammatory reactions, which could be harmful to brain tissues. Targeting and downregulation of the pyroptosis signaling pathway was an effective method to improve cognitive impairment, NLRP3 inhibitor and caspase-1 inhibitor were the most common agents to repress the activation of pyroptosis. Apart from the therapeutic methods/agents directly targeting the pyroptosis signaling pathway, different approaches have been also taken for the treatment of cognitive impairment (Table 1). Photobiomodulation (PBM) with 630 nm LED array affixed to the exposed skull of stroke mice model could significantly attenuate the progression of cognitive impairment in the chronic poststroke phase via regulating AIM2 inflammasome activation and AIM2 inflammasome-mediated pyroptosis (Kim et al., 2022). Electroacupuncture was also reported to improve 7-day survival rates and cognitive function by downregulating NLRP3/caspase-1/GSDMD pyroptosis pathway in an SAE mouse model (Li et al., 2022). Behavioral therapy such as intermittent fasting could also significantly inhibit overactivated proptosis and improve cognitive impairment (Poh et al., 2021). These attempts suggest the therapeutic effect of the non-pharmaceutical intervention and broaden the way to develop new therapy methods. The pathological changes of cognitive impairment were accompanied by the activation of the components of pyroptosis pathway, indicating the detection of pyroptosis components could be an early diagnostic biomarker. Therefore, pyroptosis played a crucial role in cognitive dysfunction, further research would be remarkably helpful for the prevention and protection of cognitive impairment.

**Table 1:** Summary of potential agents/methods targeting pyroptosis to treat cognitive impairment.

Disease	Agents/methods	Targets	Model	Ref
Alzheimer’s disease (AD)/amnestic mild cognitive impairment (aMCI)	–	NLRP3/Caspase-1/GSDMD axis	5xFAD mice	Rui et al. (2021)
	Salidroside	NLRP3 inflammasome	AD mouse model induced by Aβ1-42 and D-galactose (D-gal)/AlCl_3_, respectively	Cai et al. (2021)
	Jiedu-Yizhi formula	NLRP3/Caspase-1/GSDMD axis and LPS/Caspase-11/GSDMD pyroptosis pathways	AD rat model induced by Aβ25–35	Wang et al. (2022)
	VX765	NLRP3/Caspase-1/GSDMD axis and AIM2 activation	AD mouse model induced by sevoflurane	Tian et al. (2021)
	HC067047 or knockdown of hippocampal TRPV4	Canonical and noncanonical pyroptosis	A mouse model induced by systemic administration of lipopolysaccharide (LPS)	Guo et al. (2023)
	Verapamil/TXNIP silencing	RAGE-TXNIP axis/NLRP3 inflammasome	5xFAD mice	Sbai et al. (2022)
	Dendrobium Nobile Lindl. Alkaloid	NLRP3-Mediated Pyroptosis	AD mouse model induced by hippocampus injection of LPS	Li et al. (2022)
	Quinones	NLRP3 inflammasome	–	Chen et al. (2020)
	Schisandrin	NLRP1 inflammasome	APP/SP1 double transgenic mice	Li et al. (2021)
	N-salicyloyl tryptamine	NLRP3/Caspase-1/GSDMD axis	AD rat model induced by Aβ25–35	Bai et al. (2021)
	MCC950	NLRP3 inflammasome	SAMP8 mice	Li et al. (2020)
Postoperative cognitive dysfunction (POCD)/Perioperative Neurocognitive Disorders (PND)	Necrosulfonamide (NSA) and N-acetylcysteine (NAC)	NLRP3/Caspase-1 pathway	–	Zhou et al. (2023)
	Overexpression of DUSP14	NLRP3/Caspase-1 Pathway	A POCD aged rat model induced by isoflurane	Que et al. (2020)
	Esketamine	STING/TBK1 pathway/Caspase-1-mediated pyroptosis	A POCD aged rat model induced by sevoflurane	Li et al. (2022)
	Knockdown of SETD7	NLRP3 inflammasome	A POCD mouse model induced by isoflurane	Ma et al. (2022)
	Silencing of PINK1 and/or Ac-DEVD-CHO	Caspase-3/GSDME-Dependent Pyroptosis	A POCD rat model induced by isoflurane	Wang et al. (2023)
	microRNA-140-3p	DNMT1 mediated HTR2A/ERK/Nrf2 axis	A POCD rat model induced by sevoflurane	Wu et al. (2022)
	Elamipretide	NLRP3/Caspase-1 Pathway	A PND mouse model induced by isoflurane	Zuo et al. (2020)
	VRT-043198	NGF and BNDF expression	An aged abdominal exploratory laparotomy (AEL) mouse model of PND	Tang et al. (2022)
	MCC950	NLRP3 inflammasome	A POCD mouse model induced by isoflurane	Fan et al. (2018)
	BAY11-7082	NF-κB-mediated pyroptosis	SD rat pups at postnatal day 6 received sevoflurane	Dai et al. (2021)
	Glycyrrhizin	HMGB1-mediated NLRP3/ASC inflammasome	Pregnant rats on gestational day 20 received sevoflurane	Shan et al. (2023)
Sepsis-Associated Encephalopathy (SAE)	MCC950 & Ac-YVAD-CMK	NLRP3/Caspase-1 pathway	A mouse model of SAE induced by cecal ligation and puncture (CLP)	Fu et al. (2019)
	VX765	NLRP3/Caspase-1 pathway	A mouse model of SAE induced by CLP	Xu et al. (2019)
	Erbin	IRE1α/Xbp1s-Ca2+ axis/NLRP3 inflammasome	A mouse model of SAE induced by CLP	Jing et al. (2022)
	Dexpramipexole	Mitochondria-mediated NLRP3/caspase-1 pyroptosis	A mouse model of SAE induced by peripheral administration of lipopolysaccharide (LPS)	Zhang et al. (2023)
	Sevoflurane	Inflammatory-pyroptotic signaling (NLRP3, caspase 1/11, GSDMD, TLR4 and TRIF)	A mouse model of SAE induced by CLP	Chen et al. (2022)
	HU308	Neuronal pyroptosis	A mouse model of SAE induced by CLP	Yang et al. (2022)
	Electroacupuncture	NLRP3/Caspase-1 Pathway	A mouse model of SAE induced by CLP	Li et al. (2022)
	Recombinant club cell protein(rCC16) and/or U46619	NLRP3/Caspase-1 Pathway	A rat model of SAE induced by CLP	Zhou et al. (2019)
Chronic post-stroke cognitive impairment (PSCI)	Ac-YVAD-CMK	AIM2 inflammasome	Middle cerebral artery occlusion (MCAO)/reperfusion-induced PSCI mouse model	Kim et al. (2020)
Cognitive decline after focal cortical infarction	VX765	NLRP3-dependent pyroptosis	Distal middle cerebral artery occlusion (dMCAO) rat model	Dong et al. (2022)
Cerebral ischemia/reperfusion injury	CY-09	Caspase-1/GSDMD-dependent pyroptosis	A focal cerebral ischemia mouse model accomplished by the endovascular MCAO	Sun et al. (2020)
Ischemic stroke	Photobiomodulation	AIM2 inflammasome	Photothrombotic cortical ischemia mouse model induced by photothrombosis of the cortical microvessels	Kim et al. (2022)
	Valproic acid (VPA)	Caspased-1/NLRP1 and NLRP3 inflammasome	Ischemia/reperfusion (I/R) mouse model	Zhu et al. (2019)
Chronic cerebral hypoperfusion (CCH)/vascular dementia/vascular cognitive impairment (VCI)	Curcumin	NLRP3-dependent pyroptosis	A rat model of diabetes mellitus and CCH	Zheng et al. (2021)
	Knockout of legumain	Pyroptosis	Right unilateral common carotid artery occlusion (rUCCAO) rat model	Chai et al. (2021)
	Emodin	NLRP3 inflammasome-mediated pyroptosis	BV2 cells/HT22 cells	Jiang et al. (2023)
	AIM2 knockout	AIM2 inflammasome	Bilateral common carotid artery stenosis (BCAS) mouse model	Poh et al. (2021)
	AIM2 knockout	AIM2 inflammasome	Bilateral common carotid artery stenosis (BCAS) mouse model	Poh et al. (2021)
	Intermittent fasting (IF)	Inflammasome-associated apoptotic and pyroptotic death	Bilateral common carotid artery stenosis (BCAS) mouse model	Poh et al. (2021)
High-fat diet (HFD)-induced cognitive impairment	miR-129 antagomir	IGF-1/GSK3β Signaling Pathway	A rat model fed with HFD	Wang et al. (2021)
	miR-124	TLR4/MyD88/NF-κB p65/NLRP3 Signaling Pathway	BV2 cells treated with palmitic acid	Yang et al. (2022)
	Exogenous IGF-1	NLRP3/Caspase-1 Pathway	A mouse model fed with HFD	Sui et al. (2021)
Diabetes-associated cognitive decline	–	NLRP3 inflammasome	A diabetic rat model induced by streptozotocin (STZ)	Ruan et al. (2022)
	Naofucong	P2X7/NLRP1/Caspase-1 Pathway	HT22 cells treated with high glucose medium	Jing et al. (2021)
	Isorhynchophylline	Spliced form of X-box binding protein 1 (sXBP1)/pyroptosis	A diabetic mouse model induced by STZ	Wang et al. (2023)
Cognitive disorders of offspring from mothers with diabetes in pregnancy	Chemerin	NLRP3/Caspase-1 pathway	A diabetic pregnant mouse model induced by STZ	Liang et al. (2019)
Post-stroke cognitive impairment in diabetes	MCC950	NLRP3 inflammasome	A diabetic rat model induced by HFD/STZ, stoke rat model induced by 90-min mechanical middle cerebral artery occlusion (MCAO) surgery	Ward et al. (2019)

Recent studies also focused on the relationship between mutation of gasdermin genes and specific diseases. Ruan et al. (2018) analyzed the structure of the GSDMA3 membrane pore with the help of cryo-electron microscopy and partly revealed mechanisms of how the cleavage of GSDMA3 formed membrane pores and the mechanisms of autoinhibition. Due to a loss of autoinhibition, disease-related mutations of GSDMA3 and its N terminal alone could initiate pyroptosis and had a close association with spontaneous alopecia and hyperkeratosis (Ding et al., 2016). Single nucleotide polymorphisms (SNPs) in GSDMA and GSDMB were reported to be related to childhood asthma and to a lesser extent to adult asthma (Zheng et al., 2020). Mutations of GSDME and DFNB59 could both induce deafness, but the mechanisms were different. GSDME mutations resulted in its overexpression and led to pyroptosis in HeLa cells (Wang et al., 2017), while DFNB59 mutations exerted function in a non-pyroptosis way (Delmaghani et al., 2006). Xia et al. (2021) reported the cryo-electron microscopy structures of the pore of GSDMD, elucidating the process of GSDMD-dependent membrane pore formation and GSDMD-mediated release of IL-1β. Liu et al. (2019) revealed the mechanisms of autoinhibition, lipid binding and oligomerization of GSDMD-N-terminal in virtue of the crystal structures. These studies of molecular structure gave an explanation for the actions of a number of mutant gasdermin family members (Ruan et al., 2018). However, the association between mutation of gasdermin genes and cognitive impairment required future exploration.

Given the unknown mechanisms of cognitive impairment, we are beginning to understand the molecular biological/pathological functions of cognitive impairment and pyroptosis. Further research towards elucidating new mechanisms of the pyroptosis signaling pathway will deepen our comprehension of cognitive impairment, and provide new ideas for developing more potent methods.

## Author contributions

XY carried out the primary literature search, drafted, and revised the manuscript. ZT contributed to the drafting and revising of the manuscript. All authors contributed to the article and approved the submitted version.

## Conflict of interest

The authors declare that the research was conducted in the absence of any commercial or financial relationships that could be construed as a potential conflict of interest.

The reviewer DW declared a past co-authorship with the author ZT to the handling editor.

## Publisher’s note

All claims expressed in this article are solely those of the authors and do not necessarily represent those of their affiliated organizations, or those of the publisher, the editors and the reviewers. Any product that may be evaluated in this article, or claim that may be made by its manufacturer, is not guaranteed or endorsed by the publisher.
